# Life and health satisfaction in the adult population of Iran

**DOI:** 10.4178/epih.e2016047

**Published:** 2016-11-03

**Authors:** Rajabali Daroudi, Arash Rashidian, Hojjat Zeraati, Alireza Oliyaeemanesh, Ali Akbari Sari

**Affiliations:** 1 Department of Health Management and Economics, School of Public Health, Tehran University of Medical Sciences, Tehran, Iran; 2 Department of Epidemiology and Biostatistics, Tehran University of Medical Sciences, Tehran, Iran; 3 National Institute for Health Research, Tehran University of Medical Sciences, Tehran, Iran

**Keywords:** Subjective well-being, Life, Health, Personal satisfaction, Iran

## Abstract

**OBJECTIVES:**

Increasing interest has emerged in the use of subjective well-being as a development indicator and for the evaluation of public policies. The aim of this study was to assess life and health satisfaction and their determinants in the adult population of Iran.

**METHODS:**

We conducted a survey of a sample of 3,150 adults at least 18 years of age in Tehran, the capital of Iran. The subjects were selected using a stratified random sampling method, and they were interviewed face-to-face at their usual residence by trained interviewers. Life satisfaction was used as a measure of subjective well-being. We used ordinary least square regression models to assess the associations of life and health satisfaction with socio-demographic variables.

**RESULTS:**

On a 0-10 scale, the mean (standard deviation) scores for life and health satisfaction were 6.93 (2.54) and 7.18 (1.97), respectively. The average score for life satisfaction in females was 0.52 points higher than in males. A U-shaped relationship was found between age and life satisfaction, with respondents 35 to 44 years of age having the lowest average level of life satisfaction. Satisfaction with life and health among divorced respondents was significantly lower than among never-married and married participants. The scores for life satisfaction in respondents who rated their health status as poor were 3.83 points lower than in those who rated their health status as excellent.

**CONCLUSIONS:**

The majority of the population of Tehran was satisfied with their life and health. Self-rated health status had the greatest impact on life satisfaction.

## INTRODUCTION

In recent years, efforts have been made to incorporate the concept of subjective well-being (SWB) into policy-making. National and international surveys of SWB have been carried out in developed countries, especially in European countries. These countries have tried to use SWB data to inform policy debates and for the evaluation of public policies [1-4]. Furthermore, economists have used SWB data to measure consumer preferences and social welfare [5]. The Commission on the Measurement of Economic Performance and Social Progress (also known as the Stiglitz Commission), which was established by the French government in 2009, recommended that to assess and monitor the progress of societies, measures of SWB must be taken into account in addition to current economic performance measures such as gross domestic product [4]. Following the Stiglitz Commission recommendations, national statistical agencies in many countries, such as the UK, the US, France, Japan, and Korea have started to measure SWB. More recently, the Organization for Economic Cooperation and Development considered the nature and drivers of SWB and developed guidelines and tools for measuring it [6].

SWB indicators can be used for the appraisal and evaluation of policies in various sectors, such as health care, public health, and social services [2]. For example, policymakers can use SWB data in decision-making about resource allocation. In public health and health care, interventions are evaluated based on their benefits, which are usually measured as quality-adjusted life years (QALY). However, due to some limitations in the use of QALY, some economists have recently suggested that the benefits of health interventions could be measured using patients’ SWB [7-10]. SWB consists of three separate components: (1) the presence of positive affect, (2) the relative lack of negative affect, and (3) people’s cognitive evaluations of their life circumstances (i.e., life satisfaction or happiness) [11,12]. Positive affect denotes pleasant moods and emotions (e.g., joy, affection, interest, and engagement). Conversely, negative affect refers to the presence of unpleasant moods and emotions (e.g., anxiety and worry, anger, sadness, stress, frustration, guilt and shame, and envy). Life satisfaction or happiness is the degree to which a respondent evaluates or judges his or her life taken as a whole. Positive and negative affects taken together constitute the affective component of SWB, while life satisfaction constitutes the cognitive component [1,2,12].

Although it is necessary to measure both the affective and cognitive components in order to provide a complete picture of SWB, most previous studies have only measured the cognitive component (life satisfaction) and used it as a proxy for overall well-being. This was done because life satisfaction is a less ambiguous and more stable notion than positive and negative affect [1,12-14]. In order to measure SWB, many previous studies used single-item measures. These measures comprise questions such as, “All things considered, how satisfied are you with your life as a whole these days?” or “Taken all together, how would you say things are these days? Would you say that you are very happy, pretty happy, or not too happy?” [5,6].

Previous studies have shown relationships between SWB and other individual, social, and economic determinants. For example, studies have shown that income, education, health status, and social contact have positive effects on life satisfaction, while unemployment is negatively associated with life satisfaction [6, 15-17]. However, health is one of the most influential factors on SWB. Cross-sectional and longitudinal studies have shown a strong relationship between self-perceived health and life satisfaction [9,18-24]. Measures of SWB have been found to be correlated with both physical and mental health. Graham et al. [18] examined the effects of various health conditions on happiness. They found that anxiety and pain had greater effects on happiness than other health conditions. Furthermore, the negative effects of health conditions on happiness were very large compared to the effects of income on happiness. In another study, Böckerman et al. [9] assessed the effects of having a chronic health condition on SWB. Their study showed that all chronic health conditions had a statistically significant negative effect on SWB, and that having a psychiatric disorder had the greatest negative effect on SWB. In a study conducted by Benyamini et al. [19], a significant amount of variance in life satisfaction was explained by self-rated general health and self-rated oral health. Helliwell [20] assessed the effects of individual and societal variables on life satisfaction. The results of his study showed that an individual’s self-assessed state of health had a larger effect on life satisfaction than other socioeconomic variables. A 1-point improvement in health on a 5-point scale was associated with a 0.61-point increase in life satisfaction on a scale of 1-10. Likewise, Gerdtham & Johannesson [21] examined the relationship between happiness and socioeconomic variables in a random sample of the Swedish adult population. They found that health status had a highly significant positive effect on happiness.

Although most previous studies have assessed the effect of health on SWB, some evidence indicates that there is a two-way relationship between health and SWB. Studies have shown that well-being improves physical health outcomes and the immune system response, increases longevity and pain tolerance, and reduces the speed of disease progression [25-27]. Additionally, healthy people have higher levels of life satisfaction, while people with depression and anxiety have lower levels of well-being [28,29]. All of this evidence indicates that the measurement of SWB is very important for public policy-making, and especially for health policy-making. However, unlike in high-income countries, SWB is not measured regularly in low-income and middle-income countries such as Iran, and studies on SWB in these countries are rare. In this study, we aimed to measure life and health satisfaction and their determinants in a representative sample of the adult population of Tehran, the capital city of Iran, with populations from all ethnic and socioeconomic groups in the country.

## MATERIALS AND METHODS

### Survey

This study was based on a survey of 3,150 adults at least 18 years of age in Tehran, the capital of Iran, conducted from October 2015 to March 2016. We selected respondents using a stratified probability sampling method. The target population was stratified by municipality, and from each stratum, a random sample with a size proportionate to the population was drawn. To select respondents, each stratum (municipality region) was divided into several blocks. Then, based on the sample size from each stratum, the required number of blocks for data collection was randomly selected. In each block, 10 households were randomly invited for the interview. Families unwilling to participate in the study were replaced by new ones on a random basis. Subjects were interviewed face-to-face at their usual residence by trained interviewers.

### Questionnaire

The questionnaire collected information on sex, age, educational attainment, marital status, employment status, the presence of any illness or health problem, self-rated health status, life satisfaction, and health satisfaction. Age and educational attainment were measured in years and other personal characteristics were measured using categorical questions. The presence of any illness or health problem was assessed with the question: “Do you have any illness, health problem, condition, or disability?”. Health status was also measured by a categorical measure. In the categorical health rating question, individuals rated their own current health status on a 5-point scale (excellent, very good, good, fair, or poor). We used life satisfaction as a measure of subjective well-being. Life and health satisfaction were measured by asking: “Overall, how satisfied are you with your life (health)?” on a 0-10 scale, where 0 was not satisfied at all and 10 was completely satisfied. The validity of the questionnaire was confirmed by experts.

### Statistical methods

We performed univariate and multiple regression analyses to assess the associations of life and health satisfaction with the other variables discussed above. We created two multiple regression models. In model 1, life satisfaction was used as the dependent variable, while health satisfaction was used in model 2. The ordinary least square estimator was used to estimate the models. The Breusch-Pagan heteroscedasticity test was used to test constant variances for residuals. The Breusch-Pagan test showed heteroscedasticity in the regression models; therefore, robust standard errors were estimated for all variables to adjust for heteroscedasticity. Additionally, functional misspecification in multiple models was evaluated using the Ramsey regression equation specification error test (RESET). The significance level of 5% was used in all statistical analyses. We used Stata version 13.0 (StataCorp., College Station, TX, USA) for all analyses.

### Ethical issues

This study was approved by the ethics committee of the Deputy of Research and Technology of the Tehran University of Medical Sciences (IR.TUMS.REC.1394.743). The participants were assured that their information would remain confidential.

## RESULTS

Table 1 shows descriptive statistics for the study variables. The response rate was 70%. The mean (standard deviation [SD]) age of participants was 43.86 (15.55) years and the mean duration of schooling was 10.81 (4.75) years. Approximately 36.2 percent of participants had an illness or health problem; however, approximately 28.4 percent reported fair or poor health status. On a 0-10 scale, the mean (SD) of life satisfaction and health satisfaction were 6.93 (2.54) and 7.18 (1.97), respectively.

The results of univariate and multiple regression analysis of the associations between life satisfaction and socio-demographic variables are shown in Table 2. In the univariate analysis, life satisfaction was significantly associated with all explanatory variables. In the multiple analysis, however, the overall amount of variance explained was not high (adjusted R2=0.15), and life satisfaction was associated with all explanatory variables except with educational attainment and employment status. Other things being equal, on a 0-10 scale, the average score of life satisfaction in females was 0.52 points higher than that of males. A U-shaped relationship was found between age and life satisfaction, and the average level of life satisfaction in middle-aged respondents was lower than that of younger and older respondents. The lowest average level of life satisfaction was found in respondents 35 to 44 years of age. The average score for life satisfaction in this age group was 0.55 points lower than in the reference age group (18 to 24 years).

Married respondents reported higher average scores for life satisfaction than never-married (β=0.70) respondents, and satisfaction with life among divorced or separated respondents was significantly lower than among never-married participants (β= -0.90) (Table 2).

On average, the score for life satisfaction in respondents without health problems was 0.34 points higher than that of respondents who had a health problem. Likewise, self-rated health status had a highly significant positive effect on life satisfaction. On average, the score for life satisfaction in respondents who rated their health status as poor was 3.83 points lower than for those who rated their health status as excellent. Furthermore, the results showed a nonlinear relationship between self-rated health status and life satisfaction, such that a drop from fair to poor health was associated with a larger decrease in satisfaction with life than other 1-point downward movements in health status. Being in excellent rather than very good health added a further 0.37 points to life satisfaction, while being in poor rather than fair health decreased life satisfaction by 1.69 points. Furthermore, this nonlinear relationship was confirmed by the results of the Ramsey RESET test. As shown in Table 2, the Ramsey RESET test was significant (p<0.01) in the multiple regression model, whereas when we omitted the self-rated health status variable from the model, the *p*-value of the test was 0.54 (Table 2).

As was the case for life satisfaction, health satisfaction was significantly associated with all explanatory variables in the univariate analysis. In the multivariate analysis, health satisfaction was associated with age, marital status, and having a health problem. A negative association was found between health satisfaction and age. The age group of 75+ years had the lowest level of health satisfaction, and the health satisfaction score in that age group was 0.80 points lower than in the reference age group (18 to 24 years). On a 0-10 scale, the average scores for health satisfaction in divorced or separated respondents were 1.01 points lower than were reported by never-married respondents. On average, the score for health satisfaction in respondents without health problems was 1.39 points higher than that of respondents who had a health problem (Table 3).

## DISCUSSION

In this study, we assessed life and health satisfaction and their determinants in the adult population of the capital city of Iran. The mean (SD) scores for life and health satisfaction were 6.93 (2.54) and 7.18 (1.97), respectively. Significant associations were found among both life and health satisfaction and demographic and socioeconomic factors. In particular, self-rated health status had the greatest impact on life satisfaction.

In a study conducted in a nationally representative sample in Iran in 2006, life satisfaction was reported to be very poor in 3.9% of respondents, poor in 12.1%, moderate in 23.9%, good in 39.7%, and very good in 20.5% [30]. In our study, the level of life satisfaction was less than five on a 0-10 scale in approximately 13.0% of participants. These findings indicate that the majority of Iranian people are satisfied with their life. However, it seems that the percentage of dissatisfied people has been relatively stable between 2006 and 2015.

Our multiple analysis showed that females were more satisfied with life than males. However, no significant differences were found between the sexes in terms of satisfaction with health. These findings are consistent with previous studies [6,21,30-32]. Grant et al. [32] assessed the relationship between life satisfaction and health behaviors in young adults from 21 countries. They found that in European countries and the US, males were more satisfied with their life than females, while in Asian countries, females were more satisfied with their life than males.

We found a U-shaped relationship between age and life satisfaction. In our sample, the age group of 35 to 44 years had the lowest average level of life satisfaction. Previous studies have shown a U-shaped relationship between age and life satisfaction in high-income English-speaking countries, with the average lowest levels of well-being around ages 45 to 54. However, this pattern is not universal, and in lower-income and middle-income countries, life satisfaction has been found to decline with age [33-35]. Although the reasons for this U-shaped pattern in high-income countries has not been precisely explained, higher well-being in older ages has been explained to some extent by the theory of socioemotional selectivity. According to this theory, as people grow older, they accumulate emotional wisdom that leads to the selection of more emotionally satisfying events, friendships, and experiences [35]. Our findings indicated a similar relationship between age and life satisfaction in Iran as in English-speaking high-income countries.

In our univariate analysis, positive associations were found between education and life and health satisfaction. However, in the multivariate analysis, when controlling for additional variables, these associations were attenuated. These results correspond with those of previous studies [36,37]. This evidence indicates that education may have an impact on life and health satisfaction partially through its impact on other intermediate variables [6].

Our univariate analysis found that unemployed participants had the lowest level of life satisfaction. Other studies have found unemployment to be negatively associated with life satisfaction [16,24,30,31]. However, in our study, the association between employment status and life satisfaction was significant only in the univariate analysis. A number of studies have shown a significant association between self-perceived health and employment status [1,38,39]. Consequently, employment status may impact life satisfaction indirectly through self-perceived health.

Our results confirm findings from previous studies suggesting that married people are more satisfied with life than never-married or previously married people [20,24,30,31]. In this study, divorced or separated participants had the lowest level of life satisfaction, and the average score for life satisfaction in these participants was 1.60 points lower than in married people. The marriage-to-divorce ratio has decreased in Iran from 16.0 in 1993 to 4.4 in 2014. In addition, the age at first marriage has increased [40]. These findings indicate that by implementing policies that remove barriers to marriage and encourage young people to marry earlier, as well as policies that reduce the divorce rate, the subjective well-being of Iranian society could be increased.

We found that having any illness or health problem decreased both life and health satisfaction. These findings are consistent with previous studies that found that a chronic health condition had a statistically significant negative effect on SWB [9,18].

We found that self-rated health status had the greatest impact on life satisfaction. Furthermore, the association between self-rated health status and life satisfaction was nonlinear. The high positive impact of health status on life satisfaction has been well documented in previous studies [9,18-22]. A nonlinear relationship between self-rated health status and life satisfaction has been found in some previous studies. The result of a study conducted by Helliwell [20] showed that being in good health rather than very good health resulted in a 0.52-point decrease in subjective well-being on a 10-point scale, while being in very poor rather than poor health decreased subjective well-being by 0.46 points.

In this study, we assessed life and health satisfaction in a relatively large representative sample from the capital of Iran. However, our study had some limitations. First, we used a single-item measure to evaluate life satisfaction. Although the reliability and validity of single-item measures have been confirmed and several studies have used single-item measures, other studies have shown the reliability of multiple-item measures to be better than that of single-item measures [6]. Second, similarly to many previous studies, we measured only the cognitive component of SWB (i.e., life satisfaction) instead of measuring all three components of SWB.

In conclusion, although in recent years high-income countries have come to consider SWB as an important development indicator and started to measure SWB at the national level regularly, in low-income and middle-income countries such as Iran, knowledge about SWB remains scarce and this topic has received little attention. The results of our study showed that the majority of the population of Tehran was satisfied with their life and health, and that self-rated health status had the greatest impact on life satisfaction. Therefore, improving the health status of the population could significantly increase the SWB of society. However, we used a single-item measure to measure SWB and measured only the cognitive component of SWB. We suggest that the Iranian National Center of Statistics should develop national guidelines and tools for measuring SWB and measure it regularly at the national level. Furthermore, we suggest that researchers in Iran use multiple-item measures in future research and measure all three components of SWB.

## Figures and Tables

**Table 1.** Descriptive statistics for all variables (n = 3,150)

Variable	n	%
Life satisfaction (mean±SD)	6.93±2.54	
Health satisfaction (mean±SD)	7.18±1.97	
Years of schooling (mean±SD)	10.8±4.7	
Sex		
Male	1,556	49.4
Female	1,594	50.6
Age (yr)	330	10.5
18-24	722	22.9
25-34	658	20.9
35-44	614	19.5
45-54	499	15.8
55-64	228	7.2
65-74	100	3.2
75+	330	10.5
Employment status		
Employed	1,122	35.6
Homemaker	1,270	40.3
Retired	407	12.9
Unemployed	120	3.8
Student	230	7.3
Marital status		
Never married	574	18.2
Married	2,431	77.8
Widowed	106	3.4
Divorced or separated	39	1.2
Presence of any illness or health problem		
Yes	1,140	36.2
No	2,010	63.8
Self-rated health status		
Excellent	258	8.2
Very good	468	14.9
Good	1,530	48.6
Fair	772	24.5
Poor	122	3.9

**Table 2.** Univariate and multiple regression analysis of the associations between life satisfaction and socio-demographic variables (n = 3,150)

Explanatory variables	Univariate regression			Multiple regression		
	Coefficient		Robust SE	Coefficient		Robust SE
Sex						
Male		Reference			Reference	
Female	0.37^**^		0.09	0.52^**^		0.14
Age (yr)						
18-24		Reference			Reference	
25-34	-0.39^**^		0.15	-0.30		0.20
35-44	-0.67^**^		0.16	-0.55^*^		0.22
45-54	-0.94^**^		0.16	-0.40		0.23
55-64	-0.67^**^		0.17	-0.03		0.24
65-74	-0.71^**^		0.21	0.06		0.28
75+	-1.03^**^		0.28	0.02		0.34
Years of schooling	0.04^**^		0.01	0.02		0.01
Employment status						
Employed		Reference			Reference	
Homemaker	0.32^**^		0.10	0.13		0.16
Retired	0.08		0.15	0.09		0.18
Unemployed	-0.41^*^		0.20	-0.24		0.26
Student	0.85^**^		0.16	0.36		0.22
Marital status						
Never married		Reference			Reference	
Married	0.16		0.12	0.70^**^		0.17
Widowed	-0.39		0.28	0.47		0.30
Divorced or separated	-1.79^**^		0.47	-0.90^*^		0.45
Presence of any illness or health problem						
Yes		Reference			Reference	
No	0.94^**^		0.10	0.34^**^		0.10
Self-rated health status						
Excellent		Reference			Reference	
Very good	-0.31^*^		0.18	-0.37^*^		0.18
Good	-1.07^**^		0.16	-1.11^**^		0.17
Fair	-2.12^**^		0.18	-2.14^**^		0.19
Poor	-3.92^**^		0.32	-3.83^**^		0.34
Constant				7.15^**^		0.27
Adjusted R^2^					0.15	
F statistics					23.60^**^	
Breusch-Pagan					54.36^**^	
Ramsey RESET					4.20^**^	

SE, standard error; RESET, regression equation specification error test.

* p<0.05;

** p<0.01.

**Table 3.** Univariate and multiple regression analysis of the associations between health satisfaction and socio-demographic variables (n = 3,150)^1^

Explanatory variables	Univariate regression			Multiple regression		
	Coefficient		Robust SE	Coefficient		Robust SE
Sex						
Male		Reference			Reference	
Female	-0.33^**^		0.07	0.07		0.10
Age (yr)						
18-24		Reference			Reference	
25-34	-0.63^**^		0.11	-0.40^**^		0.13
35-44	-0.98^**^		0.11	-0.56^**^		0.15
45-54	-1.40^**^		0.12	-0.73^**^		0.15
55-64	-1.47^**^		0.13	-0.68^**^		0.17
65-74	-1.47^**^		0.16	-0.57^**^		0.20
75+	-1.83^**^		0.23	-0.80^**^		0.26
Years of schooling	0.06^**^		0.01	0.00		0.01
Employment status						
Employed		Reference			Reference	
Homemaker	-0.50^**^		0.08	-0.22		0.12
Retired	-0.46^**^		0.11	0.16		0.13
Unemployed	-0.24		0.20	0.26		0.20
Student	0.79^**^		0.11	0.11		0.14
Marital status						
Never married		Reference			Reference	
Married	-0.73^**^		0.08	-0.07		0.11
Widowed	-1.73^**^		0.24	-0.48		0.25
Divorced or separated	-1.80^**^		0.34	-1.01^**^		0.30
Presence of any illness or health problem						
Yes		Reference			Reference	
No	1.57^**^		0.07	1.39^**^		0.08
Constant				6.92^**^		0.18
Adjusted R^2^					0.17	
F statistics					38.25^**^	
Breusch-Pagan					105.44^**^	
Ramsey RESET					1.62	

SE, standard error; RESET, regression equation specification error test.

1 Since self-rated health status and health satisfaction are almost the same variables, the association between these variables was not assessed.

** p<0.01.

## References

[1] Garrido S, Méndez I, Abellán JM (2013). Analysing the simultaneous relationship between life satisfaction and health-related quality of life. J Happiness Stud.

[2] Diener E (2006). Guidelines for national indicators of subjective well-being and ill-being. J Happiness Stud.

[3] Centre for the Study of Living Standards (2011). New directions for intelligent government in Canada: papers in honour of Ian Stewart. http://www.csls.ca/festschrift/StewartFestschrift.pdf.

[4] Stiglitz JE, Sen A, Fitoussi JP (2009). The measurement of economic performance and social progress revisited: reflections and overview. https://www.ofce.sciences-po.fr/pdf/dtravail/WP2009-33.pdf.

[5] Kahneman D, Krueger AB (2006). Developments in the measurement of subjective well-being. J Econ Perspect.

[6] Aldousari S, Kassouf W (2010). Update on the management of non-muscle invasive bladder cancer. Can Urol Assoc J.

[7] Richardson J, Chen G, Khan MA, Iezzi A (2015). Can multi-attribute utility instruments adequately account for subjective well-being?. Med Decis Making.

[8] Dolan P, Metcalfe R (2012). Valuing health: a brief report on subjective well-being versus preferences. Med Decis Making.

[9] Böckerman P, Johansson E, Saarni SI (2011). Do established health-related quality-of-life measures adequately capture the impact of chronic conditions on subjective well-being?. Health Policy.

[10] Wang X, Jia X, Zhu M, Chen J (2015). Linking health states to subjective well-being: an empirical study of 5854 rural residents in China. Public Health.

[11] Diener Ed, Suh E, Oishi S (1997). Recent findings on subjective well-being. Indian J Clin Psychol.

[12] Arthaud-Day ML, Rode JC, Mooney CH, Near JP (2005). The subjective well-being construct: a test of its convergent, discriminant, and factorial validity. Soc Indic Res.

[13] Helliwell JF, Putnam RD (2004). The social context of well-being. Philos Trans R Soc Lond B Biol Sci.

[14] Blanchflower DG, Oswald AJ (2004). Well-being over time in Britain and the USA. J Public Econ.

[15] Sacks DW, Stevenson B, Wolfers J (2010). Subjective well-being, income, economic development and growth. http://www.nber.org/papers/w16441.pdf.

[16] Winkelmann L, Winkelmann R (1998). Why are the unemployed so unhappy? Evidence from panel data. Economica.

[17] Dolan P, Peasgood T, White M (2008). Do we really know what makes us happy? A review of the economic literature on the factors associated with subjective well-being. J Econ Psychol.

[18] Graham C, Higuera L, Lora E (2011). Which health conditions cause the most unhappiness?. Health Econ.

[19] Benyamini Y, Leventhal H, Leventhal EA (2004). Self-rated oral health as an independent predictor of self-rated general health, self-esteem and life satisfaction. Soc Sci Med.

[20] Helliwell JF (2003). How’s life? Combining individual and national variables to explain subjective well-being. Econ Model.

[21] Gerdtham UG, Johannesson M (2001). The relationship between happiness, health, and socio-economic factors: results based on Swedish microdata. J Socio Econ.

[22] Kwan YK (2010). Life satisfaction and self-assessed health among adolescents in Hong Kong. J Happiness Stud.

[23] Sabatini F (2014). The relationship between happiness and health: evidence from Italy. Soc Sci Med.

[24] Sun S, Chen J, Johannesson M, Kind P, Burström K (2016). Subjective well-being and its association with subjective health status, age, sex, region, and socio-economic characteristics in a Chinese population study. J Happiness Stud.

[25] Shields MA, Price SW (2005). Exploring the economic and social determinants of psychological well-being and perceived social support in England. J R Stat Soc Ser A Stat Soc.

[26] Howell RT, Kern ML, Lyubomirsk S (2007). Health benefits: meta-analytically determining the impact of well-being on objective health outcomes. Health Psychol Rev.

[27] Diener E, Chan MY (2011). Happy people live longer: subjective well-being contributes to health and longevity. Appl Psychol Health Well Being.

[28] Keyes CL (2005). Mental illness and/or mental health? investigating axioms of the complete state model of health. J Consult Clin Psychol.

[29] Haller M, Hadler M (2006). How social relations and structures can produce happiness and unhappiness: an international comparative analysis. Soc Indic Res.

[30] Mousavi M, Shiani M, Mohammadi MA, Sadjadi H, Tabatabaee F, Assari S (2011). Life satisfaction in Iran: a national representative study. Sci Res Essays.

[31] Strine TW, Chapman DP, Balluz LS, Moriarty DG, Mokdad AH (2008). The associations between life satisfaction and health-related quality of life, chronic illness, and health behaviors among U.S. community-dwelling adults. J Community Health.

[32] Grant N, Wardle J, Steptoe A (2009). The relationship between life satisfaction and health behavior: a cross-cultural analysis of young adults. Int J Behav Med.

[33] Blanchflower DG, Oswald AJ (2008). Is well-being U-shaped over the life cycle?. Soc Sci Med.

[34] Deaton A (2008). Income, health, and well-being around the world: evidence from the Gallup World Poll. J Econ Perspect.

[35] Steptoe A, Deaton A, Stone AA (2015). Subjective wellbeing, health, and ageing. Lancet.

[36] Blanchflower DG, Oswald AJ (2011). International happiness: a new view on the measure of performance. Acad Manage Perspect.

[37] Helliwell JF (2008). Life satisfaction and quality of development. http://www.nber.org/papers/w14507.pdf.

[38] Al-Windi A (2005). The relations between symptoms, somatic and psychiatric conditions, life satisfaction and perceived health. A primary care based study. Health Qual Life Outcomes.

[39] Eikemo TA, Bambra C, Judge K, Ringdal K (2008). Welfare state regimes and differences in self-perceived health in Europe: a multilevel analysis. Soc Sci Med.

[40] Gholipour HF, Farzanegan MR (2015). Marriage crisis and housing costs: Empirical evidence from provinces of Iran. J Policy Model.

